# Individualised decision making: interpretation of risk for extremely preterm infants—a survey of UK neonatal professionals

**DOI:** 10.1136/archdischild-2021-322147

**Published:** 2021-08-19

**Authors:** Katherine Wood, Lydia Mietta Di Stefano, Helen Mactier, Sarah Elizabeth Bates, Dominic Wilkinson

**Affiliations:** 1 Department of Newborn Care, John Radcliffe Hospital, Oxford, UK; 2 Faculty of Medicine, Nursing and Health Sciences, Monash University, Clayton, Victoria, Australia; 3 Department of Neonatology, Princess Royal Maternity, Glasgow, UK; 4 School of Medicine, University of Glasgow, Glasgow, UK; 5 Department of Women & Childrens, Great Western Hospitals NHS Foundation Trust, Swindon, UK; 6 Uehiro Centre for Practical Ethics, University of Oxford, Oxford, UK

**Keywords:** ethics, neonatology, resuscitation

## Abstract

**Background:**

The British Association of Perinatal Medicine (BAPM) published a revised framework for perinatal management of extremely preterm infants (EPIs) in 2019. We aimed to assess UK neonatal professionals’ interpretation of elements of this framework, as well as the consistency of their estimates of outcome for EPIs.

**Methods:**

An online survey gave participants five cases involving anticipated extremely preterm birth with different favourable and unfavourable risk factors. Respondents were asked to assign a risk category and management option using the BAPM framework and to estimate the chance of survival if the baby received active resuscitation and the chance of severe disability if they survived.

**Results:**

Respondents were consistent in interpretation of risk categories. The majority would follow parental wishes about management. Management decisions did not always correspond with risk assessment, with less inclination to recommend palliative (comfort) care. There were wide estimates of survival or severe disability (5%–90%) with consultants providing lower estimates of severe disability than other groups.

**Conclusion:**

UK neonatal professionals deferred to parental wishes in the cases presented, indicating an emphasis on shared decision making. However, they did not necessarily use the risk stratification approach for management decisions. Variation in estimates of outcome raises questions about the accuracy of informed decision making and suggests support is needed for UK clinicians to incorporate risk factors into individualised counselling. There may be value in validating existing online risk calculators for UK infants or in developing a UK specific risk model.

## Abstract (precis)

Topical and important insight into the current state of play in the UK regarding active management at the limit of viability.

**What is already known on this topic?**

- British Association of Perinatal Medicine (BAPM) published a revised framework for perinatal management of extremely preterm infants (EPIs) in 2019.
- The revised BAPM framework encourages professionals to assess the risk for individual infants and to base management decisions on the assessed risk in conjunction with parents.
- Previous studies have reported variations in professionals’ estimates of survival and severe disability for EPIs.

**What this study adds?**

- Neonatal professionals’ interpretation of the BAPM framework risk assessment is generally consistent, but subsequent management decisions do not always follow recommendations from the framework.
- UK neonatal professionals are likely to be guided by parental views when making decisions about perinatal management of EPIs.
- UK neonatal professionals’ estimates of survival and severe disability for EPIs vary widely, with implications for the accuracy of informed shared decision making.

## Introduction

Guidance relating to perinatal care of extremely preterm infants (EPIs) aims to support professionals and parents faced with difficult ethical decisions. Consistency of advice must be balanced with the need to tailor decisions to individual circumstances.^1^

**Figure 1:**
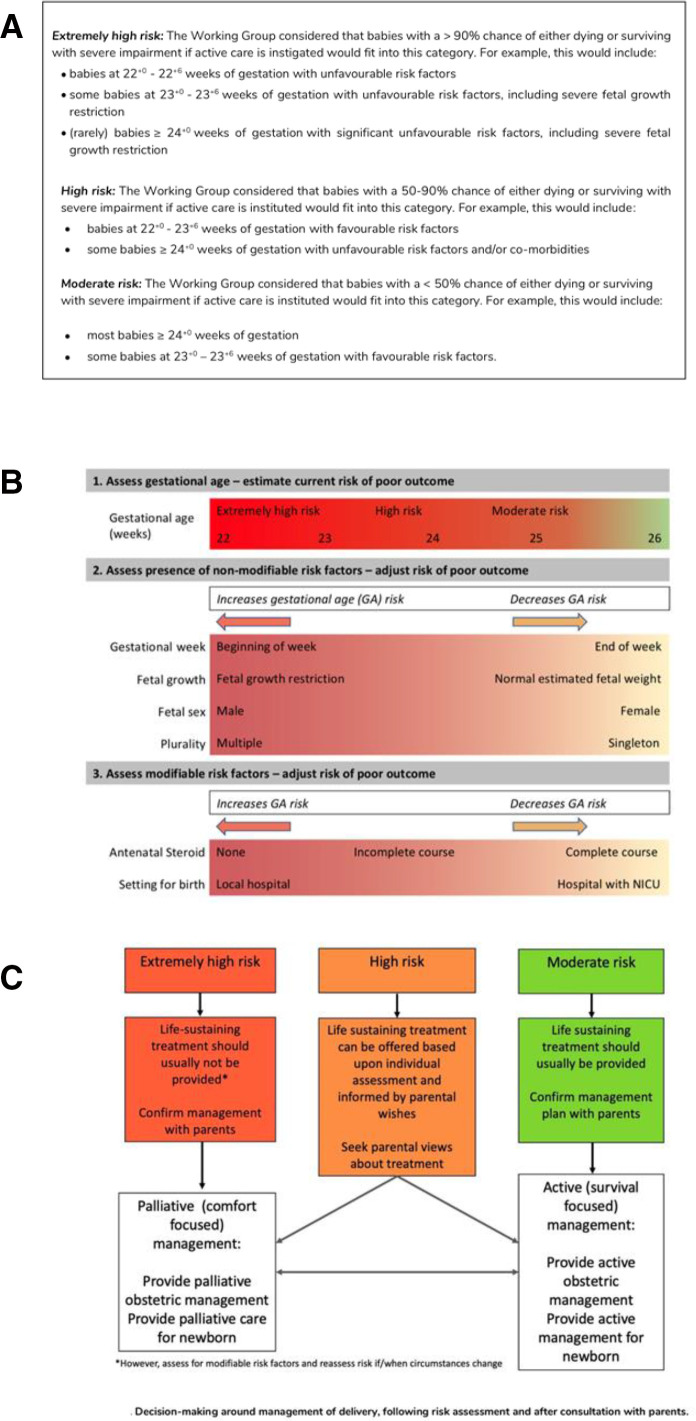
Figures from the BAPM framework provided for reference in the survey. (A) Consensus for risk categorisation, (B) visual tool for refinement of risk, (C) flow diagram for the decision making around management of delivery. Figure reproduced from ref 2 with permission. BAPM, British Association of Perinatal Medicine.

The British Association of Perinatal Medicine (BAPM) published a revised framework for the perinatal management of EPI in 2019.^2^ This framework attempts to move away from gestation-based decisions and encourages a risk assessment taking into account gestational age, fetal growth, sex, plurality, antenatal steroids and birth location. The aim is to stratify the risk of a poor outcome into ‘extremely-high’, ‘high’ or ‘moderate’, corresponding to a chance of dying or surviving with severe disability of >90%, 50%–90% and <50%, respectively (figure 1A). These risk categories can be used to guide the perinatal management. For infants at ‘extremely-high risk’, palliative (comfort-focused) care would be recommended, while for those at ‘moderate risk’, the recommendation would be active (survival focused) care. For infants at ‘high risk’, the decision should be based on parents’ wishes (figure 1C).^2^

Encouraging involvement of parents in shared decision making is an important aspect of the BAPM framework. Parents can find information about the likelihood of survival and neurodevelopmental impairment useful when making decisions about the care for their baby.^3 4^ Therefore, accurate estimates are needed. The BAPM framework provides a summary of average outcome at different gestational ages, but it does not specify precisely how estimates should be adjusted to take into account other factors.

We have previously reported a shift in the approach of UK neonatal professionals to EPI since the publication of the revised framework, with a widening of the ‘grey zone’ for decision making.^5^ It is not known how UK neonatal professionals interpret the framework’s risk assessment, nor how consistent are their estimates of the chances of survival and/or severe disability.

## Methods

An anonymous online survey was developed to assess UK neonatal clinicians’ attitudes and interpretation of the BAPM framework and distributed in mid-2020. The methods of the wider survey have been described previously.^5^ Participants were three groups of UK neonatal professionals involved in antenatal decision making around management of EPIs; consultants, middle grade trainee doctors (‘registrars’) and advance neonatal nurse practitioners (ANNPs).

### Design

To evaluate risk assessment, respondents were given five clinical scenarios in random order involving anticipated EPI birth with different favourable and unfavourable risk factors (table 1). In each case, an early dating scan had been performed, and there were no other medical conditions affecting the fetus or mother. To aid recall, respondents were provided with figures from the BAPM framework (figure 1).

**Table 1:** Case scenarios for risk assessment

Case letter and brief summary (abbreviation used in text)	Description of case as provided to respondents in the survey	Summary of case			Authors’ interpretation of risk categorisation
		Gestational age	Favourable risk factors	Unfavourable risk factors	
A 23+3 weeks, favourable risk factors (23+3F)	A mother has gone into extremely preterm labour at 23+3 weeks’ gestation. She is in a hospital with a NICU and has received a full course of steroids. The fetus is a singleton, has normal growth (estimated fetal weight of 590 g) and is known to be female.	23+3 midweek	NICU Normal Growth Steroids Female Singleton		High to moderate
B 25+0 weeks, unfavourable risk factors (25+0U)	A mother has gone into extremely preterm labour at 25+0 weeks’ gestation, with a twin pregnancy. She is currently in a local hospital and has had no steroids. Both twins are male and have growth restriction (estimated fetal weight of twin 1 is 520 g and estimated fetal weight of twin 2 is 560 g).	25+0 Beginning of week		Local hospital Growth restriction No steroids Male Twins	High
C 22+6 weeks, favourable risk factors (22+6F)	A mother has gone into extremely preterm labour at 22+6 weeks’ gestation. She is currently in a hospital with a NICU and has had a full course of steroids. The male fetus is a singleton and has normal growth (estimated fetal weight 600 g).	22+6 End of week	NICU Normal growth Steroids Singleton	Male	High
D 22+3 weeks, favourable risk factors (22+3F)	A mother has gone into extremely preterm labour at 22+3 weeks’ gestation. She is currently in a hospital with a NICU and has received a full course of steroids. The fetus is a singleton, normally grown. (estimated fetal weight 500 g) and is known to be female.	22+3 Midweek	NICU Normal growth Steroids Singleton Female		Extremely high to high
E 23+4 weeks, unfavourable risk factors (23+4U)	A mother has gone into extremely preterm labour at 23+4 weeks gestation. She is currently in a local hospital and has had no steroids. The fetus is a singleton, with growth restriction (estimated fetal weight 450 g) and is known to be male.	23+4 Midweek	Singleton	Local hospital Growth restriction No steroids Male	Extremely high

Details of cases as provided in the survey, a summary of the favourable and unfavourable risk factors (not provided to respondents) and authors’ interpretation of risk category according to the BAPM framework (not provided to respondents). Red text indicates unfavourable risk factors, orange text indicates intermediate risk and green text indicates favourable risk factors. Each case has been given an abbreviation (used further in the main text) of the gestational age followed by U or F, depending whether there is a majority of unfavourable (U) or favourable (F) risk factors.

NICU, neonatal intensive care unit.

For each scenario, respondents were asked to assign a risk category and allocate a management option using the BAPM framework. Respondents were asked if they agreed with this management. If they indicated palliative or active care would be recommended, they were asked whether they would offer the opposite on parents’ request.

Respondents were asked to estimate the chance of survival if the baby received active resuscitation and the chance of severe disability if the baby survived (to nearest 5%). See online supplemental table 1 for full text of questions and details on ethical approval.

10.1136/fetalneonatal-2021-322147.supp1 Supplementary data

### Analysis

Consenting respondents who answered more than one question were included in the analysis.

Data are presented as percentages and medians with IQRs. McNemar’s test, χ^2^ test, paired t-test and Mann Whitney U test were used to compare responses. Statistical significance was set at 0.05. Comparisons between groups used Bonferroni correction. For analyses of data and statistics, we used Microsoft Excel 2019 and GraphPad Prism V.9.0.0.

## Results

### Respondents

There were 336 eligible responses. Consultants formed the highest proportion (50%). The majority of respondents worked in a neonatal intensive care unit (NICU) (75%) and were aged 31–40 years. Sixty-eight per cent were female and 40% had more than 16 years’ experience working with EPI (online supplemental table 2).^5^ Ninety-five per cent reported having read the revised BAPM framework and 89% indicated that they were currently using it in their clinical practice.

### Risk categorisation

**Figure 2:**
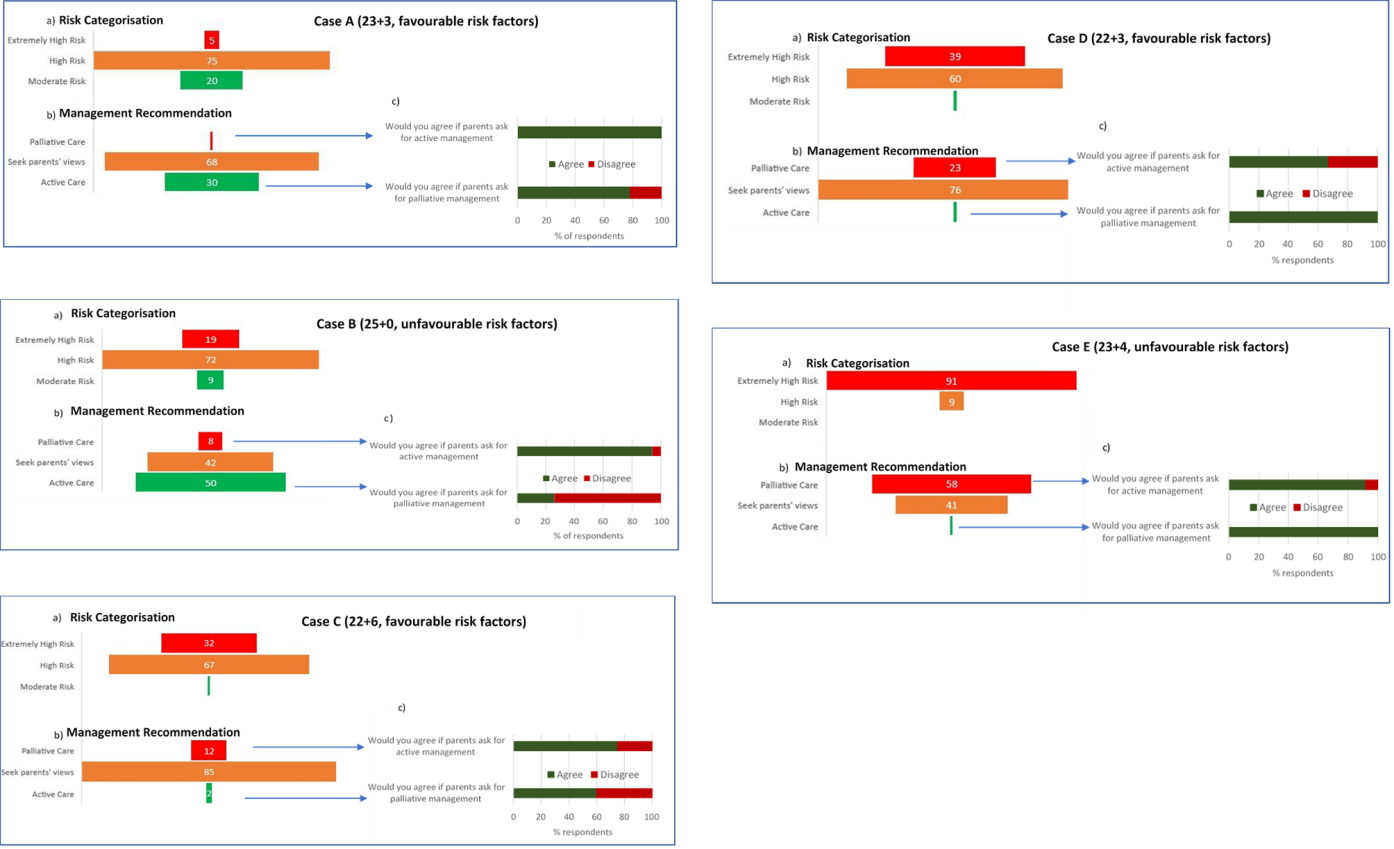
(A) Percentage of respondents that categorised each case as ‘extremely high’, ‘high’ and ‘moderate’ risk using the BAPM framework; (B) the percentage of respondents who allocated management for each case as palliative (comfort focused), seek parents’ views or active (survival focused) using the BAPM framework. (C) Bar chart showing percentage of respondents who would agree to the opposite management they allocated in figure part B on parent’s request. BAPM, British Association of Perinatal Medicine.

Each case has been given an abbreviation of gestational age followed by U or F, depending whether there is a majority of unfavourable (U) or favourable (F) risk factors. The respondents’ risk categorisations are illustrated in figure 2A. The majority (60%–75%) classified risk as ‘high’ for all cases except case E (23+4 unfavourable (23+4U)), where 91% classified it as ‘extremely-high’. For cases B (25+0U), C (22+6 favourable (22+6F)) and D (22+3F), respondents were divided between ‘extremely-high’ and ‘high’ risk. Case A (23+3F) had the highest proportion of ‘moderate’ risk classification at 20%.

### Perinatal management

Management decisions are indicated in figure 2B. The majority of respondents (68%–85%) indicated that they would seek parents’ views about decisions in cases A (23+3F), C (22+6F) and D (22+3F). In case B (25+0U), 50% would plan active treatment, while in case E (23+4U), 58% would plan palliative care. In most cases, respondents advising active or palliative care would be prepared to provide the alternate management if parents insisted, including case E (23+4U) (92% of those who would advise palliative care would nevertheless provide active treatment if requested). The exception was in case B (25+0U) where only 29% of respondents would agree to palliative care when advising active treatment (figure 2C).

In all cases, more respondents categorised the risk as ‘extremely-high’ than allocated the management as palliative care (online supplemental table 3). For case E (23+4U), 202 (91%) respondents stratified it as ‘extremely-high’ risk, but only 37% of these respondents indicated they would provide palliative care (p<0.001). Conversely in cases A (23+3F) and B (25+0U) (the lower risk cases), a significantly higher proportion advised active treatment than categorised the risk as moderate (case B; advise active treatment; n=111 vs categorise moderate risk; n=19 p<0.001)).

More than 85% supported the management that they interpreted the BAPM framework as recommending. An exception was case B (25+0U) where 23% (n=21) who interpreted the framework as recommending seeking parents’ views did not support this.

There were some differences between professional groups and types of centre in their recommendations (online supplemental tables 4 and 5).

### Estimates of survival and severe disability

#### Survival

**Figure 3:**
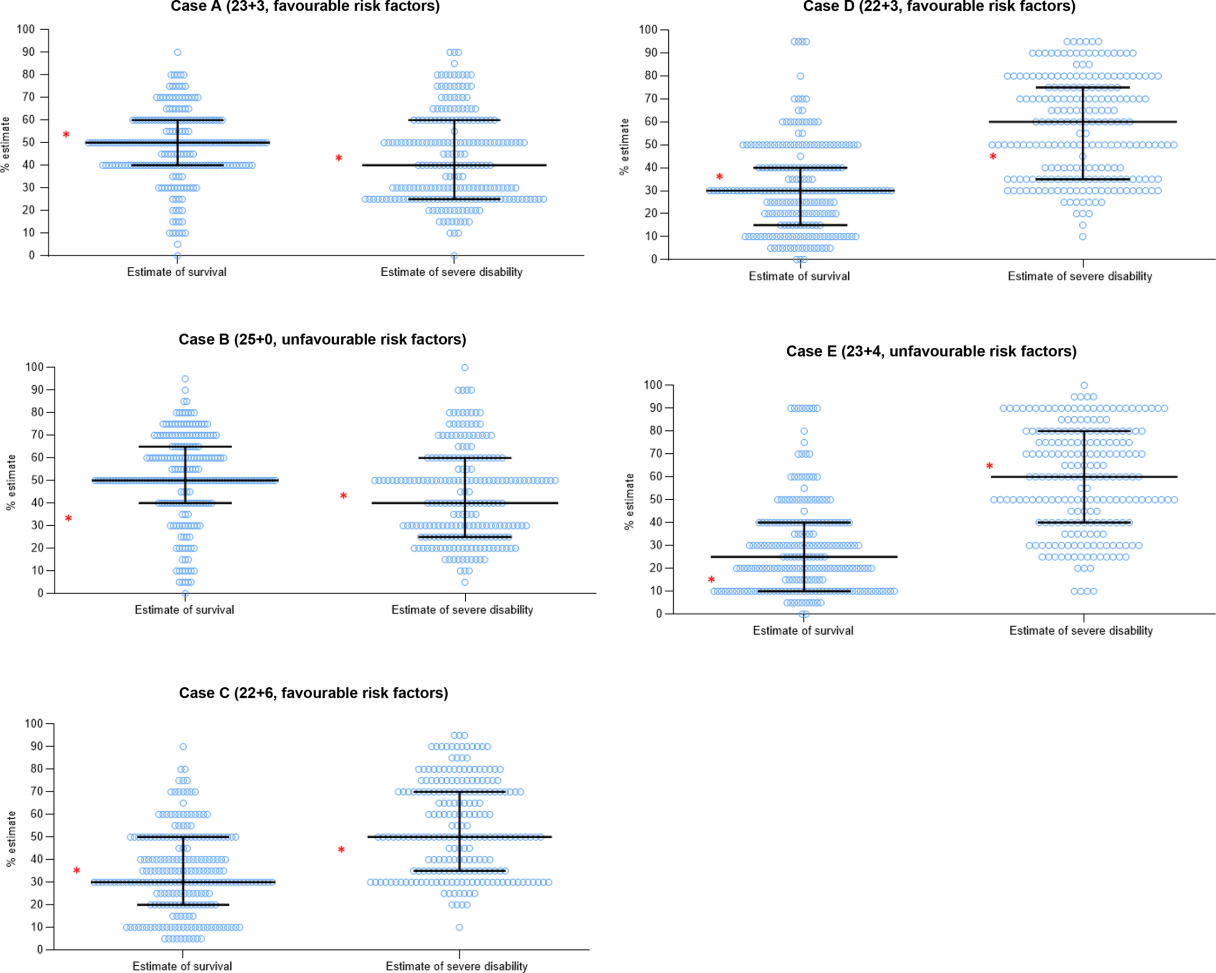
Dot plots showing respondents’ estimates of survival and severe disability in percentages. Each dot represents one respondent’s estimates. Horizontal lines represent the median and IQR. Asterisks represent the estimates of survival and the average estimate of moderate/severe neurodevelopmental impairment from the online NICHD Extremely Preterm Birth Outcomes Tool.^3^

In every case, a wide range of estimates of survival were given, with minimum and maximum estimates ranging between 5% and 90% (figure 3). The narrowest IQR of 20% was seen in case A (23+3F) and widest in cases E (23+4U) (IQR 10%–40%) and C (22+6F) (IQR 20%–50%).

There was a significant difference between the estimates given in the two cases at 22 weeks ((C 22+6F and D 22+3F) (survival t=−2.72, p=0.007 and disability t=3.6, p<0.001)) and 23 weeks ((A 23+3F and E2 3+4U) (t=14.3, p<0.001 and t=13.4, p<0.001, respectively)).

The range and median estimates of survival were similar between the professional groups. In case E (23+4U), consultants gave a lower (but not statistically significant) median estimate of survival compared with registrars and ANNPs (20% vs 30%) (online supplemental figure 1).

There was no difference between the median estimates of survival from respondents working in NICUs compared with those working in LNUs (local neonatal units) and SCUs (special care units) (online supplemental figure 2).

#### Severe disability

There was a wide range of estimates of severe disability (10%–90%) in each case (figure 3). The IQRs were comparable between the cases (35%–40%).

In all cases, consultants gave the lowest median estimate of severe disability, and ANNPs gave the highest. There was a significant difference between the estimates of consultants and ANNPs for cases B, C and D, and a significant difference between the estimates of consultants and registrars in cases B and D (online supplemental figure 3)

There was no difference seen in the estimate of severe disability between respondents working in NICUs and those working in LNU/SCUs (online supplemental figure 2).

#### Estimates of risk of dying or severe disability compared with categorisation of risk

**Figure 4:**
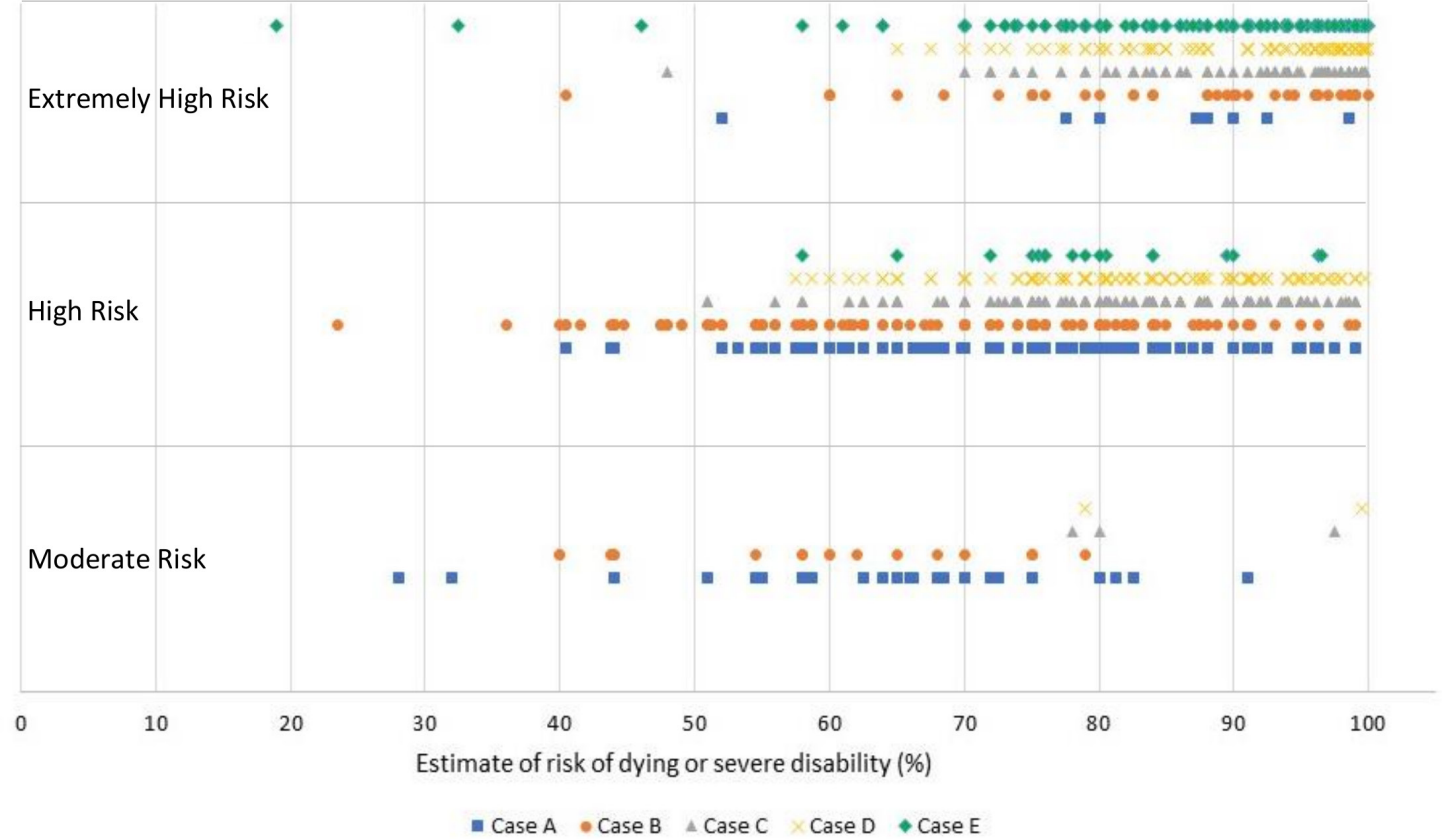
Scatter plot showing respondents categorisation of risk versus their estimate of risk of dying or severe disability. Each mark represents a respondent’s categorisation of risk and their corresponding composite estimate of risk of dying and severe disability for each given case.

Figure 4 compares the respondents’ estimates of risk of dying or severe disability compared with their categorisation of risk. Where respondents categorised a case as ‘extremely-high’, ‘high’ and ‘moderate’ risk the majority of estimates were clustered in the range of 70%–100%, 50%–99% and 45%–80%, respectively.

## Discussion

This study provides insights into how UK neonatal professionals approach risk assessment and counselling for EPI in the context of the revised BAPM framework. Given a set of cases of EPI with a mix of risk factors, respondents were relatively consistent in interpreting the risk classification and in seeking parental wishes about management. However, risk and management decisions did not always correspond. Moreover, there were very wide and variable estimates for the chance of survival or severe disability if the infant survived, with some differences between professional groups’ estimates.

### Interpretation of BAPM framework

We found a relatively high level of agreement in the classification of risk across the cases. For all cases (except for case E (23+4U)), the majority of respondents felt the risk was ‘high’.

According to the BAPM framework, where a case is categorised as ‘extremely-high’ risk, palliative care would be in the best interests of the baby and life-sustaining treatment should not be offered. Yet in the survey, the number of respondents classifying the risk as ‘extremely-high’ was significantly higher than the number of respondents recommending palliative care. In fact, it appeared that in almost all the scenarios, respondents would follow parental wishes.

These findings suggest variation in interpretation of the BAPM risk stratification approach to decision making, with a stronger deference (arguably appropriate) to parental wishes. We hypothesise that this reflects increasing emphasis towards shared decision making between professionals and parents.^4 6 7^ With the apparent widening of the ‘grey zone’ for decision making,^5^ there may be an increase in the range of cases requiring parental input.

Being guided by parental wishes where there is uncertainty about the best interests of the newborn is ethically appropriate.^1^ However, responses also potentially indicate relative discomfort in recommending palliative care.^8^ The fact that the majority of respondents would agree to active management on parental request despite identifying scenarios as ‘extremely high-risk’ could indicate that the revised framework is not sufficiently clear, or that in the setting of an individualised approach, neonatal clinicians are reluctant to decline to provide active survival focused care. This could raise concerns about the impact of the framework on infants’ best interests in some cases.

An additional reason for these observations could be the relatively short interval between publication of the framework (October 2019) and the survey (June–August 2020), as professionals may need time to understand and incorporate a more complex risk stratification model into their practice.

### Estimates of survival and severe disability

In our survey, professionals appeared to incorporate risk factors into their estimates of outcome for EPI and not base these estimates on gestational age alone.

Defining a ‘correct’ estimate of survival and severe disability is difficult.^9^ The BAPM framework provides average figures based on gestation alone. Some online calculators allow adjustment for risk factors. The US National Institute of Child Health and Human Development (NICHD) calculator bases estimates on completed weeks of gestation, fetal weight, sex, plurality and antenatal steroids.^10^ A revised 2020 version has been shown to be moderately accurate,^11^ but its applicability to infants born in the UK is unknown and does not include estimates for outborn infants. The authors are unaware of a UK equivalent.

The median estimates of survival given by participants in our survey were close to those from the NICHD calculator (<7% difference) in three cases (A (23+3F), C (22+6F) and D(22+3F)), but 15% and 10% higher in cases B (25+0U) and E (23+4U), respectively. This may be due to respondents not taking full account of the impact of unfavourable risk factors on outcomes. This finding is in contrast to studies from USA and Australia, which found that healthcare professionals underestimate survival rates for EPI^12–14^ but in agreement with a previous UK study that found professionals overestimated survival rates at 24–25 weeks’ gestation.^15^

(We could not directly compare estimates of disability from our survey with the NICHD tool as the latter provides estimates for combined moderate and severe disability as opposed to severe disability.)

One striking finding from our survey is the wide variation in the estimates of survival and disability, with estimates ranging from 5% to 90%. Similar variation has been previously reported^14 16^ and may reflect the difficulties healthcare professionals face in taking into account different risk factors without assigned numerical values, as well as variable understanding, interpretation and recall of the literature on EPI outcomes.

We saw no significant difference between professional groups or respondents working in NICUs versus LNU/SCUs in the median estimates of survival. Consultants consistently estimated a lower chance of severe disability than registrars and ANNPs; this may be because consultants are more likely to be involved in follow-up EPIs leading to a different perception of the rates of severe disability. This could also be influenced by transfer of follow-up of the most severely impaired children to community services.

Estimates of survival and disability did not clearly map on to the risk classification assigned by neonatal professionals. We found that generally respondents gave higher chance of dying or severe disability with increasing risk category, but there was a large variation of estimates in all categories, with no constraint to the suggested range in the BAPM framework.

This wide variation in estimates of survival and severe disability has significant ethical implications. While the majority of respondents would be guided by parents’ views in determining management, parents’ views may be influenced by information provided to them about potential outcomes for their baby.^6 17–19^ Our results suggest parents may be given considerably different estimates of outcome, which may ultimately affect their decisions. Discussion with different neonatal professionals therefore carries a risk of receiving conflicting information, with potential to cause added stress for expectant mothers and their partners^20^ as well as parental confusion and uncertainty.^21^

### Strengths and limitations

To our knowledge, this is the first research looking at the interpretation of the new BAPM EPI risk framework, giving us an insight into how it may be being used in UK clinical practice.

We included a spectrum of neonatal professionals making decisions on perinatal management of EPI and working across all levels of neonatal units. We acknowledge that answers based on hypothetical cases may not truly reflect what practitioners would do in clinical practice. We were unable to estimate the response rate to the survey due to cross-over from different recruitment sources (respondents could have been on more than one of the mailing lists used). As described previously, we believe that we have included around 25% of UK neonatal consultants.^5^ While this constitutes a typical response rate for an online survey, results may not be representative of the wider profession. We also only had a small proportion (2%) of respondents working in LNUs, so results may not be fully representative of this group’s views.

We invited only neonatal consultants/registrars/ANNPs to participate as these professionals are likely to be at the forefront of decision making relating to postnatal management of EPI. However, obstetricians and other members of the wider perinatal team have a crucial role to play in the management of extremely preterm birth. Future research should explore the views of obstetricians, midwives, neonatal nurses, other paediatricians and, importantly, parents.

In the survey, we did not explicitly define severe disability when asking respondents for estimates. Although the BAPM framework provides a detailed description of severe impairment,^2^ we did not assess whether respondents recalled or endorsed this. The variability observed in our survey may therefore reflect variations in definitions as well as variations in estimated prognosis.

## Conclusions

In managing EPI in the UK, there is general concordance between professionals in assessment and classification of risk, but this does not necessarily translate to management recommended within the recently updated BAPM framework. This suggests that further education is required if a risk stratification approach is used for perinatal management of EPI.

Clinicians appeared to be mostly guided by parental wishes, indicating an emphasis on shared decision making and a widening of the ‘grey zone’ for decisions.

We found a concerning degree of variation in estimates of survival and severe disability for EPI. This has potential to influence the choices parents make, raising questions about consistency in counselling and the accuracy of informed decision making and reinforces the importance of providing written information as recommended within the BAPM framework. There is a need for further training to support UK perinatal professionals in how to incorporate different risk factors into their individualised prognostication and counselling. There would also be value in validating existing online risk calculators for UK infants, or in developing a UK specific risk model, allowing professionals to generate consistent estimates that take into account multiple risk factors.

## Data Availability

Data are available in a public, open access repository.
