# Supplementary material for: Individualised decision making: interpretation of risk for extremely preterm infants—a survey of UK neonatal professionals
Source: Arch Dis Child Fetal Neonatal Ed. 2021 Aug 19;107(3):281–8. doi: 10.1136/archdischild-2021-322147 (PMC9046748; doi:10.1136/archdischild-2021-322147)
Supplement: Supplementary data [file fetalneonatal-2021-322147supp001.pdf]

**Supplementary Figures and Tables**

| | |
|---|---|
| Q4.1<br>**SECTION 2A**<br><br>Q4.2 | <br><br><br>Have you read the new *BAPM Framework for Practice on Perinatal Management of Extreme Preterm Birth before 27 weeks of gestation*?<br><br>○ Yes<br><br>○ No |
| Q4.3 | Are you using the new *BAPM Framework for Practice on Perinatal Management of Extreme Preterm Birth before 27 weeks of gestation* to guide your clinical practice?<br><br>○ Yes<br><br>○ No |
| Q4.4 | If no, why not? |
| Q4.5<br>**SECTION 2B** | We would like you to read the following scenarios and use the new *BAPM Framework for Practice on Perinatal Management of Extreme Preterm Birth before 27 weeks of gestation* to consider the risk you would assign to each case (using the information in figure 1: *Visual tool for refinement of* risk and box 1: *Consensus for risk categorisation*).<br>After assigning the risk we would like to think about what outcome you would estimate for the baby and how you would manage the case around delivery (using the information from figure 2: *Flow diagram for decision making around management of delivery*).<br><br>If you would like to review these figures, they will appear at the bottom of the screen for all of the following questions.<br>For each case, please assume that that an early dating scan has been performed to estimate gestational age, and that there are no other known medical conditions affecting the fetus or mother. |

Note that the cases will appear in random order.

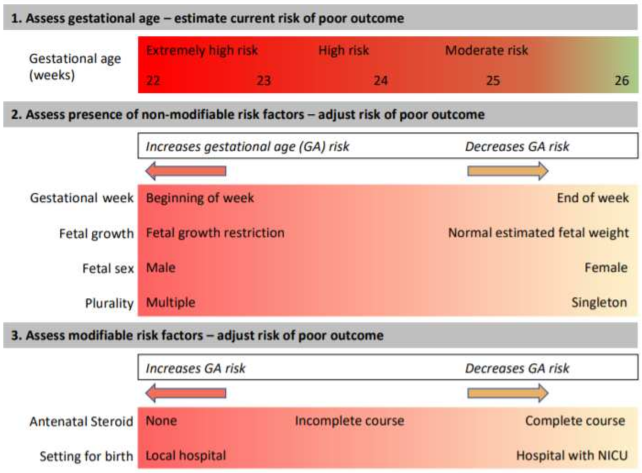


**Figure 1: Proposed visual tool for refinement of risk**

BOX 1

***Extremely high risk:*** The Working Group considered that babies with a > 90% chance of either dying or surviving with severe impairment if active care is instigated would fit into this category. For example, this would include:

- babies at $22^{+0}$ - $22^{+6}$ weeks of gestation with unfavourable risk factors
- some babies at $23^{+0}$ - $23^{+6}$ weeks of gestation with unfavourable risk factors, including severe fetal growth restriction
- (rarely) babies ≥ $24^{+0}$ weeks of gestation with significant unfavourable risk factors, including severe fetal growth restriction

***High risk:*** The Working Group considered that babies with a 50-90% chance of either dying or surviving with severe impairment if active care is instituted would fit into this category. For example, this would include:

- babies at $22^{+0}$ - $23^{+6}$ weeks of gestation with favourable risk factors
- some babies ≥ $24^{+0}$ weeks of gestation with unfavourable risk factors and/or co-morbidities

***Moderate risk:*** The Working Group considered that babies with a < 50% chance of either dying or surviving with severe impairment if active care is instituted would fit into this category. For example, this would include:

- most babies ≥ $24^{+0}$ weeks of gestation
- some babies at $23^{+0}$ – $23^{+6}$ weeks of gestation with favourable risk factors.

**Box 1 represents the consensus of the Working Group in regard to risk categories for the purposes of this framework.**

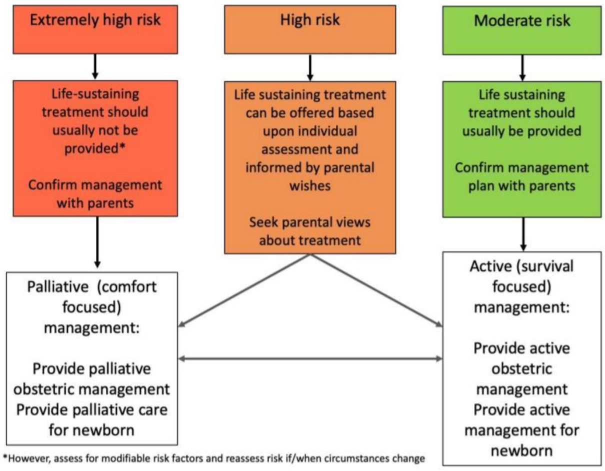


**Figure 2. Decision-making around management of delivery, following risk assessment and after consultation with parents.**

| | |
|---|---|
| Q5.1 | **Case A**; A mother has gone into extremely preterm labour at **23+3** weeks' gestation. She is in a hospital with a **NICU** and has received a full course of **steroids**. The fetus is a **singleton**, has normal growth (estimated fetal weight of **590g**) and is known to be **female**. |
| Q5.2 | Using the risk categories taken from the BAPM Framework for Practice what level of risk would you assign to this case? (Scroll down to the bottom of this screen if you would like to review the figures from the guidance).<br>◯ Extremely high risk<br>◯ High risk<br>◯ Moderate risk |
| Q5.3 | If the baby receives active resuscitation, what is your estimate for the chance of survival to discharge from hospital to the nearest 5%? |
| Q5.4 | If the baby survives to discharge, what do you think is the chance of severe disability to the nearest 5%? |
| Q5.5 | Based on your understanding of the BAPM Framework for Practice, what management is recommended in this case? (Scroll down to the bottom of this screen if you would like to review the figures from the guidance).<br>◯ Recommend palliative (comfort focused) management<br>◯ Be guided by parent's views on whether to choose palliative (comfort focused) management or active (survival focused) management<br>◯ Recommend active (survival focused) management |
| Q5.9 | You have indicated the BAPM Framework for Practice would recommend active (survival focused) management. If you were caring for this patient, would you support this management plan?<br>◯ Yes<br>◯ No |
| Q5.10 | If no, please elaborate on why: |
| Q5.11 | Would you be prepared to offer palliative (comfort focused) management at parents' request?<br>◯ Yes<br>◯ No |

| | |
|---|---|
| Q5.12 | You have indicated the BAPM Framework for Practice would recommend palliative (comfort focused) management. If you were caring for this patient, would you support this management plan?<br>○ Yes<br>○ No |
| Q5.13 | If no, please elaborate on why: |
| Q5.14 | If the parents are insistent that they wish for active resuscitation, should this be an option?<br>○ Yes<br>○ No |
| Q5.15 | You have indicated the BAPM Framework for Practice would recommend being guided by parent's views on whether to choose palliative (comfort focused) management or active (survival focused) management. If you were caring for this patient, would you support this management plan?<br>○ Yes<br>○ No |
| Q5.16 | If no, please elaborate on why: |
| Questions 5.2 -5.16 repeated for each of the following case: | |
| | Q6.1 Case D; A mother has gone into extremely preterm labour at 22+3 weeks gestation. She is currently in a hospital with a NICU and has received a full course of steroids. The fetus is a singleton, normally grown (estimated fetal weight 500g) and is known to be female. |
| | Q7.1 Case E; A mother has gone into extremely preterm labour at 23+4 weeks gestation. She is currently in a local hospital and has had no steroids. The fetus is a singleton, with growth restriction (estimated fetal weight 450g) and is known to be male. |
| | Q8.1 Case B; A mother has gone into extremely preterm labour at 25+0 weeks gestation, with a twin pregnancy. She is currently in a local hospital and has had no steroids. Both twins are male and have growth restriction (estimated fetal weight of twin 1 is 520g and estimated fetal weight of twin 2 is 560g). |

| | |
|---|---|
| | Q9.1 Case C; A mother has gone into extremely preterm labour at 22+6 weeks gestation. She is currently in a hospital with a NICU and has had a full course of steroids. The male fetus has normal growth (estimated fetal weight 600g). |

**Supplementary Table 1:** Full text of survey questions. The study was approved by the University of Oxford Central University Research Ethics Council (R69766/RE001) in June 2020 and was registered with the Monash University Human Research Ethics Committee (Ref 23763).

| Characteristic (n= number of responses) | n (%) |
|---|---|
| **Professional Role (n= 336)** | |
| Consultant | 167 (50) |
| Registrar | 109 (32) |
| ANNP | 42 (13) |
| Other | 18 (5) |
| **Years working with extremely preterm infants (n= 200)** | |
| 0-3 | 12 (6) |
| 4-7 | 57 (29) |
| 8-15 | 50 (25) |
| >16 | 81 (40) |
| **Age (years) (n=193)** | |
| 18-30 | 3 (2) |
| 31-40 | 89 (46) |
| 41-50 | 41 (21) |
| 51-60 | 51 (26) |
| 61-70 | 7 (4) |
| >71 | 2 (1) |
| **Neonatal centre usually worked in (n= 203)** | |
| NICU | 158 (78) |
| LNU | 40 (20) |
| SCU | 4 (2) |
| **Gender (n=198)** | |
| Female | 136 (68) |
| Male | 61 (31) |
| Other | 1 (1) |

**Supplementary Table 2**: Baseline characteristics of respondents.

Special Care Units (SCU): These provide special care for their own local population. They also provide, by agreement with their neonatal network, some high dependency services.

Local Neonatal Units (LNU): These provide special care and high dependency care and a restricted volume of intensive care (as agreed locally) and would expect to transfer babies who require complex or longer-term intensive care to a Neonatal Intensive Care Unit.

Neonatal Intensive Care Unit (NICU): These are larger intensive care units that provide the whole range of medical (and sometimes surgical) neonatal care for their local population and additional care for babies and their families referred from the neonatal network in which they are based, and also from other networks when necessary, to deal with peaks of demand or requests for specialist care not available elsewhere. Many will be sited within perinatal centres that are able to offer similarly complex obstetric care. These units will also require close working arrangements with all of the relevant paediatric sub-specialties.

(As referenced from British Association of Perinatal Medicine Service Standards for Hospitals Providing Neonatal Care (3rd Edition 2010)

| | **Risk categorisation (n) vs management recommendation (n) with P Value** | | |
|---|---|---|---|
| **Case** | **Extremely high vs palliative care** | **High vs Seeking Parents' Wishes** | **Moderate vs active treatment** |
| **A (23+3F)** | **12 vs 3, P=0.007** | **168 vs 153, P=0.032** | **44 vs 68, P<0.001** |
| **B (25+0U)** | **43 vs 17, P< 0.001** | **159 vs 93, P<0.001** | **19 vs 111, P<0.001** |
| **C (22+6F)** | **72 vs 28, P< 0.001** | **151 vs 193, P<0.001** | 3 vs 5, P=0.68 |
| **D (22+3F)** | **89 vs 52, P< 0.001** | **135 vs 172, P<0.001** | 2 vs 2, P=0.62 |
| **E (23+4U)** | **202 vs 128, P< 0.001** | **19 vs 91, P<0.001** | 0 vs 2, P=1 |

**Supplementary Table 3;** Number of respondents categorising extremely high, high and moderate risk versus number of respondents recommending palliative care, seeking parents' wishes and active treatment respectively. P values represent chance of difference between risk categorisation and corresponding management recommendation from the BAPM guideline using McNemar's test. Statistical significance was taken as $p<0.05$.

Each case has been given an abbreviation of the gestational age followed by U or F, depending whether there is a majority of unfavourable (U) or favourable (F) risk factors.

**Comparison of risk management between groups**

A significantly higher number of ANNPs (43%) compared to consultants (14%) and registrars/fellows (20%) classified the risk for case B (25+0U) as extremely-high (p <0.001 and 0.02 respectively). Fewer consultants (3%) recommended palliative care compared to registrars/fellows (13%) and ANNPs (13%) (p=0.08 and 0.03 respectively).

A higher proportion of respondents working in a local neonatal unit (LNU) or a special care unit (SCU) compared to those working in a NICU recommended palliative care compared to from NICUs for case C (22+6F) (25 vs 10%, p 0.007).

| **CASE A (23+3F)** | **Number (%)** | | | **$X^2$ and P value for difference between groups** | | |
|---|---|---|---|---|---|---|
| **Risk and Management Allocation** | **Consultant** | **Reg/ Fellow** | **ANNP** | **Consultant and Reg/Fellow** | **Consultant and ANNP** | **Reg/Fellow and ANNP** |
| **Extremely High** | 11 (10) | 1 (1) | 0 (0) | $X^2$ 6.2 p 0.01 | $X^2$ 0.82 p 0.36 | $X^2$ 0.88 p 0.35 |
| **High** | 79 (69) | 71 (83) | 18 (75) | $X^2$ 4.6 p 0.03 | $X^2$ 0.31 p 0.58 | $X^2$ 0.69 p 0.40 |
| **Moderate** | 24 (21) | 14 (16) | 6 (25) | $X^2$ 0.72 p 0.39 | $X^2$ 1.13 p 0.29 | $X^2$ 2.66 p 0.10 |
| | | | | | | |
| **Palliative Care** | 3 (3) | 0 (0) | 0 (0) | $X^2$ 2.3 p 0.13 | $X^2$ 0.64 p 0.42 | N/A |
| **Seek parents' wishes** | 72 (63) | 62 (72) | 19 (79) | $X^2$ 1.77 p 0.18 | $X^2$ 2.26 p 0.13 | $X^2$ 0.48 p 0.49 |
| **Active Care** | 39 (34) | 24 (28) | 5 (21) | $X^2$ 0.9 p 0.342 | $X^2$ 1.63 p 0.2 | $X^2$ 0.18 p 0.67 |

| **CASE B (25+0U)** | **Number (%)** | | | **$X^2$ and P value for difference between groups** | | |
|---|---|---|---|---|---|---|
| **Risk and Management Allocation** | **Consultant** | **Reg/ Fellow** | **ANNP** | **Consultant and Reg/Fellow** | **Consultant and ANNP** | **Reg/Fellow and ANNP** |
| **Extremely High** | 15 (14) | 18 (20) | 10 (43) | $X^2$ 1.64 p 0.2 | **$X^2$ 11 p <0.001** | $X^2$ 5.12 p 0.02 |
| **High** | 82 (75) | 65 (74) | 12 (52) | $X^2$ 0.01 p 0.91 | $X^2$ 4.6 p 0.03 | $X^2$ 4.03 p 0.04 |
| **Moderate** | 13 (12) | 5 (6) | 1 (4) | $X^2$ 2.22 p 0.14 | $X^2$ 1.13 p 0.29 | $X^2$ 0.063 p 0.08 |
| | | | | | | |
| **Palliative Care** | 3 (3) | 11 (13) | 3 (13) | $X^2$ 7.1 p 0.008 | $X^2$ 4.7 p 0.03 | $X^2$ 0.005 p 0.94 |
| **Seek parents' wishes** | 40 (36) | 43 (49) | 10 (43) | $X^2$ 3.13 p0.08 | $X^2$ 0.41 p 0.52 | $X^2$ 0.21 p 0.65 |
| **Active Care** | 67 (61) | 34 (39) | 10 (43) | $X^2$ 9.7 p 0.002 | $X^2$ 2.7 p 0.12 | $X^2$ 0.18 p 0.67 |

| **CASE C (22+6F)** | **Number (%)** | | | **$X^2$ and P value for difference between groups** | | |
|---|---|---|---|---|---|---|
| **Risk and Management Allocation** | **Consultant** | **Reg/ Fellow** | **ANNP** | **Consultant and Reg/Fellow** | **Consultant and ANNP** | **Reg/Fellow and ANNP** |
| **Extremely High** | 72 (64) | 61 (70) | 18 (69) | $X^2$ 0.9 p 0.34 | $X^2$ 0.28 p 0.60 | $X^2$ 0.007 p 0.93 |
| **High** | 39 (35) | 25 (29) | 8 (31) | $X^2$ 0.75 p 0.38 | $X^2$ 0.13 p 0.72 | $X^2$ 0.04 p 0.84 |
| **Moderate** | 2 (2) | 1 (1) | 0 (0) | $X^2$ 0.13 p 0.72 | $X^2$ 0.47 p 0.49 | $X^2$ 0.3 p 0.58 |
| | | | | | | |
| **Palliative Care** | 16 (14) | 9 (10) | 3 (12) | $X^2$ 0.65 p 0.42 | $X^2$ 0.12 p 0.73 | $X^2$ 0.03 p 0.86 |
| **Seek parents' wishes** | 93 (82) | 77 (89) | 23 (88) | $X^2$ 1.38 p 0.22 | $X^2$ 0.58 p 0.45 | $X^2$ <0.001 p 0.99 |
| **Active Care** | 4 (4) | 1 (1) | 0 (0) | $X^2$ 1.15 p 0.28 | $X^2$ 0.95 p 0.33 | $X^2$ 0.3 p 0.58 |

| **CASE D (22+3F)** | **Number (%)** | | | **$X^2$ and P value for difference between groups** | | |
|---|---|---|---|---|---|---|
| **Risk and Management Allocation** | **Consultant** | **Reg/ Fellow** | **ANNP** | **Consultant and Reg/Fellow** | **Consultant and ANNP** | **Reg/Fellow and ANNP** |
| **Extremely High** | 46 (41) | 32 (36) | 11 (46) | $X^2$ 0.64 p 0.42 | $X^2$ 0.18 p 0.67 | $X^2$ 0.85 p 0.36 |
| **High** | 66 (59) | 56 (62) | 13 (54) | $X^2$ 0.22 p 0.63 | $X^2$ 0.18 p 0.67 | $X^2$ 0.51 p 0.47 |
| **Moderate** | 0 (0) | 2 (2) | 0 (0) | $X^2$ 2.5 p 0.11 | N/A | $X^2$ 0.27 p 0.60 |
| | | | | | | |
| **Palliative Care** | 32 (29) | 13 (14) | 7 (29) | $X^2$ 5.7 p 0.02 | $X^2$ 0.003 p 0.95 | $X^2$ 2.84 p 0.09 |
| **Seek parents' wishes** | 80 (71) | 76 (84) | 16 (67) | $X^2$ 4.8 p 0.02 | $X^2$ 0.22 p 0.64 | $X^2$ 3.8 p 0.05 |
| **Active Care** | 0 (0) | 1 (1) | 1 (4) | $X^2$ 1.25 p 0.26 | $X^2$ 4.7 p 0.03 | $X^2$ 1.03 p 0.31 |

| **CASE E (23+4U)** | **Number (%)** | | | **$X^2$ and P value for difference between groups** | | |
|---|---|---|---|---|---|---|
| **Risk and Management Allocation** | **Consultant** | **Reg/ Fellow** | **ANNP** | **Consultant and Reg/Fellow** | **Consultant and ANNP** | **Reg/Fellow and ANNP** |
| **Extremely High** | 103 (93) | 77 (91) | 22 (88) | $X^2$ 0.31 p 0.58 | $X^2$ 0.63 p 0.43 | $X^2$ 0.14 p 0.70 |
| **High** | 8 (7) | 8 (9) | 3 (12) | $X^2$ 0.31 p 0.58 | $X^2$ 0.63 p 0.43 | $X^2$ 0.14 p 0.70 |
| **Moderate** | 0 (0) | 0 (0) | 0 (0) | N/A | N/A | N/A |
| | | | | | | |
| **Palliative Care** | 64 (58) | 54 (64) | 10 (40) | $X^2$ 0.69 p 0.41 | $X^2$ 2.56 p 0.11 | $X^2$ 4.4 p 0.04 |
| **Seek parents' wishes** | 45 (41) | 31 (36) | 15 (60) | $X^2$ 0.34 p 0.56 | $X^2$ 3.1 p 0.08 | $X^2$ 4.4 p 0.04 |
| **Active Care** | 2 (2) | 0 (0) | 0 (0) | $X^2$ 1.55 p 0.21 | $X^2$ 0.46 p 0.5 | N/A |

**Supplementary Table 4:** For cases A to E the number and percentage of respondents classifying the risk as extremely high, high or moderate and allocating the management as palliative care, seeking parents' wishes or active care by professional group, with the $X^2$ and P values between these estimates by professional groups, as calculated by Chi Squared Test. Statistical significance was taken as p<0.001.

Each case has been given an abbreviation of the gestational age followed by U or F, depending whether there is a majority of unfavourable (U) or favourable (F) risk factors.

| **CASE A (23+3F)** | **Number (%)** | | **$X^2$ and P value for difference between groups** |
|---|---|---|---|
| **Risk and Management Allocation** | **NICU** | **LNU/SCU** | **NICU and LNU/SCU** |
| **Extremely High** | 5 (3) | 6 (14) | $X^2$ 7.32 p 0.007 |
| **High** | 122 (77) | 30 (68) | $X^2$ 1.5 p 0.22 |
| **Moderate** | 31 (20) | 8 (18) | $X^2$ 0.04 p 0.83 |
| | | | |
| **Palliative Care** | 0 (0) | 3 (7) | **$X^2$ 10.9 p <0.001** |
| **Seek parents' wishes** | 111 (70) | 29 (66) | $X^2$ 0.31 p 0.58 |
| **Active Care** | 47 (30) | 12 (27) | $X^2$ 0.101 p 0.75 |

| **CASE B (25+0U)** | **Number (%)** | | **$X^2$ and P value for difference between groups** |
|---|---|---|---|
| **Risk and Management Allocation** | **NICU** | **LNU/SCU** | **NICU and LNU/SCU** |
| **Extremely High** | 29 (18) | 8 (18) | $X^2$ 0.002 p 0.965 |
| **High** | 112 (71) | 33 (75) | $X^2$ 0.23 p 0.63 |
| **Moderate** | 16 (10) | 3 (7) | $X^2$ 0.46 p 0.5 |
| | | | |
| **Palliative Care** | 12 (8) | 2 (5) | $X^2$ 0.51 p 0.48 |
| **Seek parents' wishes** | 60 (38) | 26 (59) | $X^2$ 6.11 p 0.01 |
| **Active Care** | 85 (54) | 16 (36) | $X^2$ 4.3 p 0.04 |

| **CASE C (22+6F)** | **Number (%)** | | **$X^2$ and P value for difference between groups** |
|---|---|---|---|
| **Risk and Management Allocation** | **NICU** | **LNU/SCU** | **NICU and LNU/SCU** |
| **Extremely High** | 47 (30) | 18 (41) | $X^2$ 0.89 p 0.17 |
| **High** | 109 (69) | 25 (57) | $X^2$ 2.46 p 0.12 |
| **Moderate** | 1 (1) | 1 (2) | $X^2$ 0.933 p 0.334 |
| | | | |
| **Palliative Care** | 15 (10) | 11 (25) | $X^2$ 7.28 p 0.007 |
| **Seek parents' wishes** | 138 (88) | 32 (73) | $X^2$ 6.1 p 0.01 |
| **Active Care** | 4 (3) | 1 (2) | $X^2$ 0.01 p 0.917 |

| **CASE D (22+3F)** | **Number (%)** | | **$X^2$ and P value for difference between groups** |
|---|---|---|---|
| **Risk and Management Allocation** | **NICU** | **LNU/SCU** | **NICU and LNU/SCU** |
| **Extremely High** | 63 (40) | 18 (41) | $X^2$ 0.009 p 0.93 |
| **High** | 93 (59) | 26 (59) | $X^2$ 0.0003 p 0.986 |
| **Moderate** | 1 (1) | 0 (0) | $X^2$ 0.28 p 0.60 |
| | | | |
| **Palliative Care** | 36 (23) | 14 (32) | $X^2$ 1.45 p 0.23 |
| **Seek parents' wishes** | 120 (76) | 30 (68) | $X^2$ 1.23 p 0.26 |
| **Active Care** | 1 (1) | 0 (0) | $X^2$ 0.28 p 0.6 |

| **CASE E (23+4U)** | **Number (%)** | | **$X^2$ and P value for difference between groups** |
|---|---|---|---|
| **Risk and Management Allocation** | **NICU** | **LNU/SCU** | **NICU and LNU/SCU** |
| **Extremely High** | 147 (94) | 38 (86) | $X^2$ 2.48 p 0.12 |
| **High** | 10 (6) | 6 (14) | $X^2$ 2.48 p 0.12 |
| **Moderate** | 0 (0) | 0 (0) | N/A |
| | | | |
| **Palliative Care** | 98 (62) | 24 (55) | $X^2$ 0.89 p 0.35 |
| **Seek parents' wishes** | 58 (37) | 20 (45) | $X^2$ 1.04 p 0.31 |
| **Active Care** | 1 (1) | 0 (0) | $X^2$ 0.29 p 0.60 |

**Supplementary Table 5:** For cases A to E the number and percentage of respondents classifying the risk as extremely high, high or moderate and allocating the management as palliative care, seeking parents' wishes or active care by centre worked in (neonatal intensive care unit (NICU) or local neonatal unit (LNU)/special care unit (SCU)), with the $X^2$ and P values between these estimates by centre worked in, as calculated by Chi Squared Test. Statistical significance was taken as $p<0.001$**.**

Each case has been given an abbreviation of the gestational age followed by U or F, depending whether there is a majority of unfavourable (U) or favourable (F) risk factors.

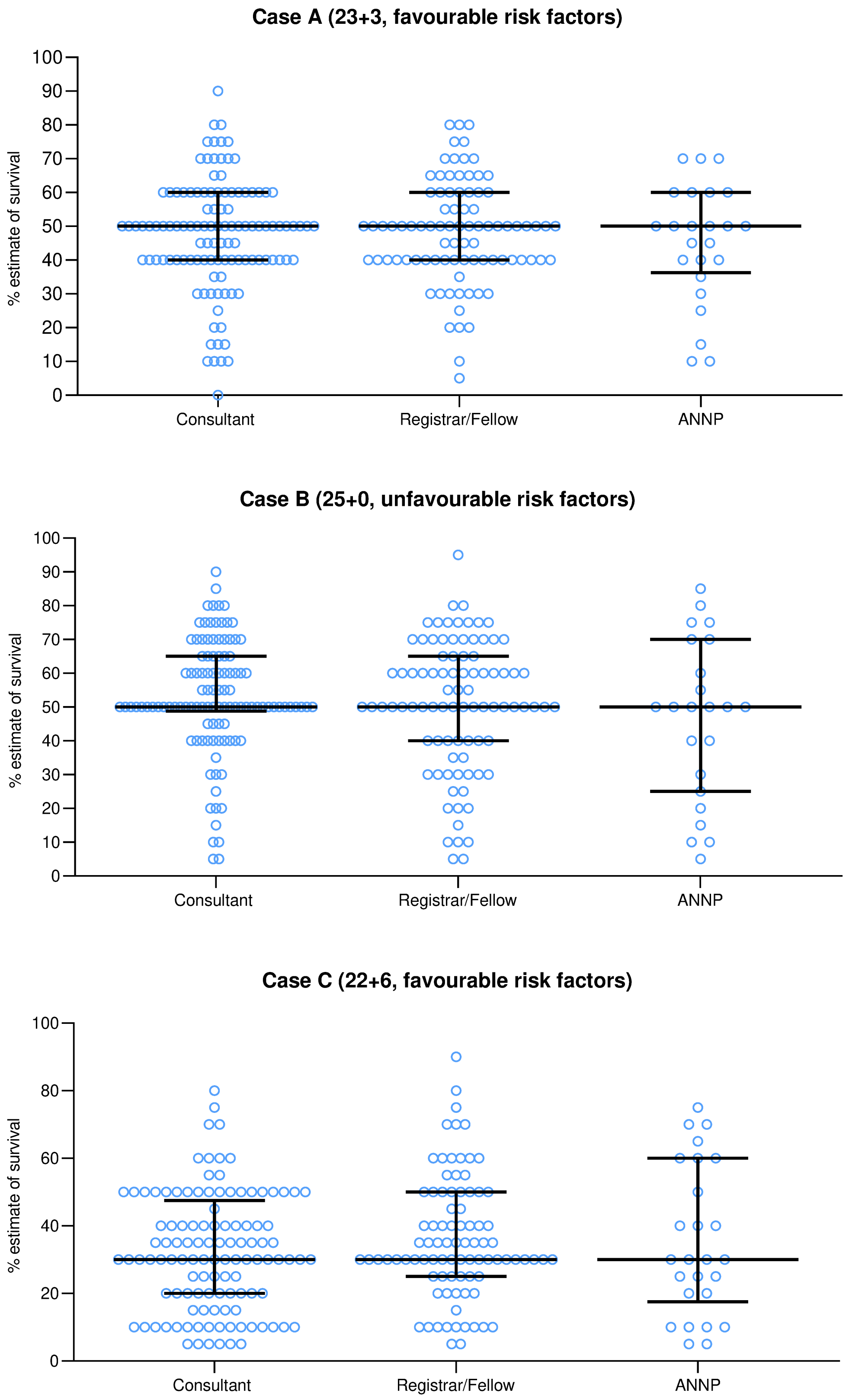
Case A (23+3, favourable risk factors)
% estimate of survival
100
90
80
70
60
50
40
30
20
10
0
Consultant
Registrar/Fellow
ANNP
Case B (25+0, unfavourable risk factors)
% estimate of survival
100
90
80
70
60
50
40
30
20
10
0
Consultant
Registrar/Fellow
ANNP
Case C (22+6, favourable risk factors)
% estimate of survival
100
80
60
40
20
0
Consultant
Registrar/Fellow
ANNP

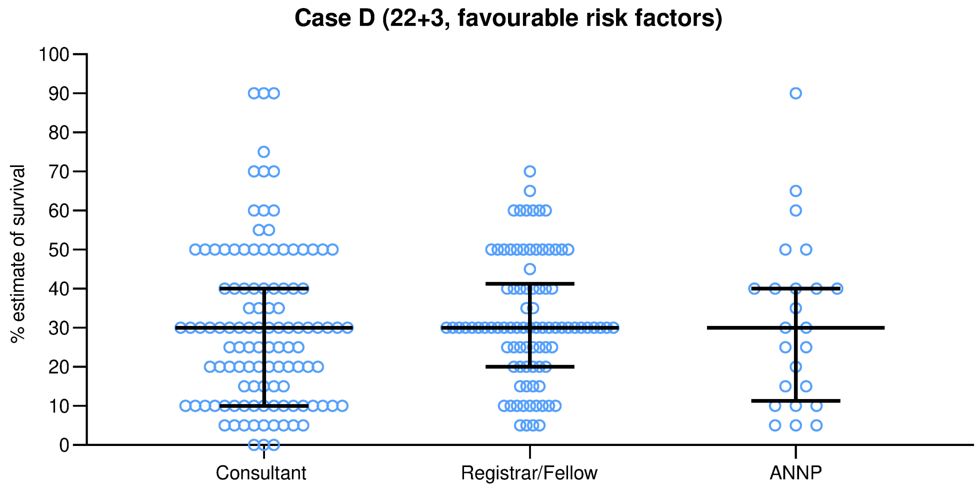


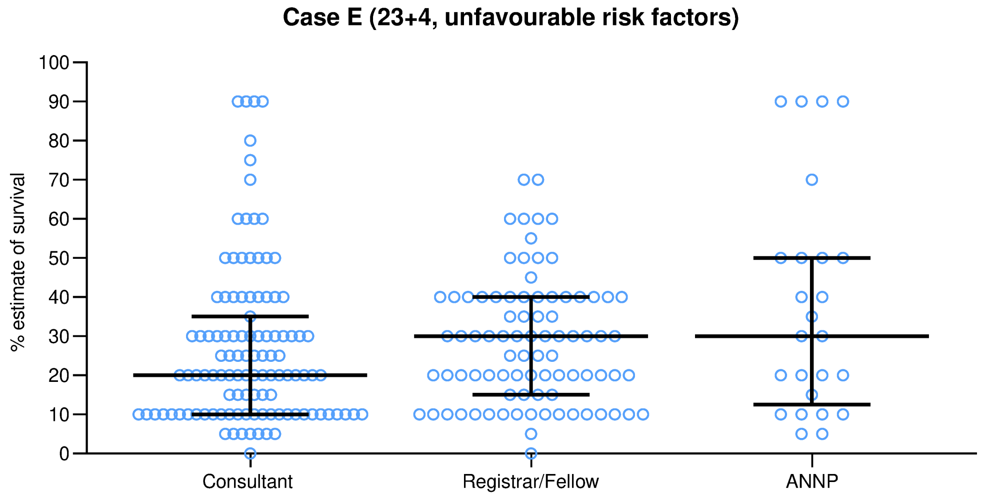


**Supplementary figure 1**: Dot plots showing respondents estimates of survival in percentages by professional group. Each dot represents one respondent's estimates. The horizontal lines represent the median and interquartile range. There was no statistical difference found between the different professional group's estimates (using Mann Whitney U test. Statistical significance was taken as p<0.005)

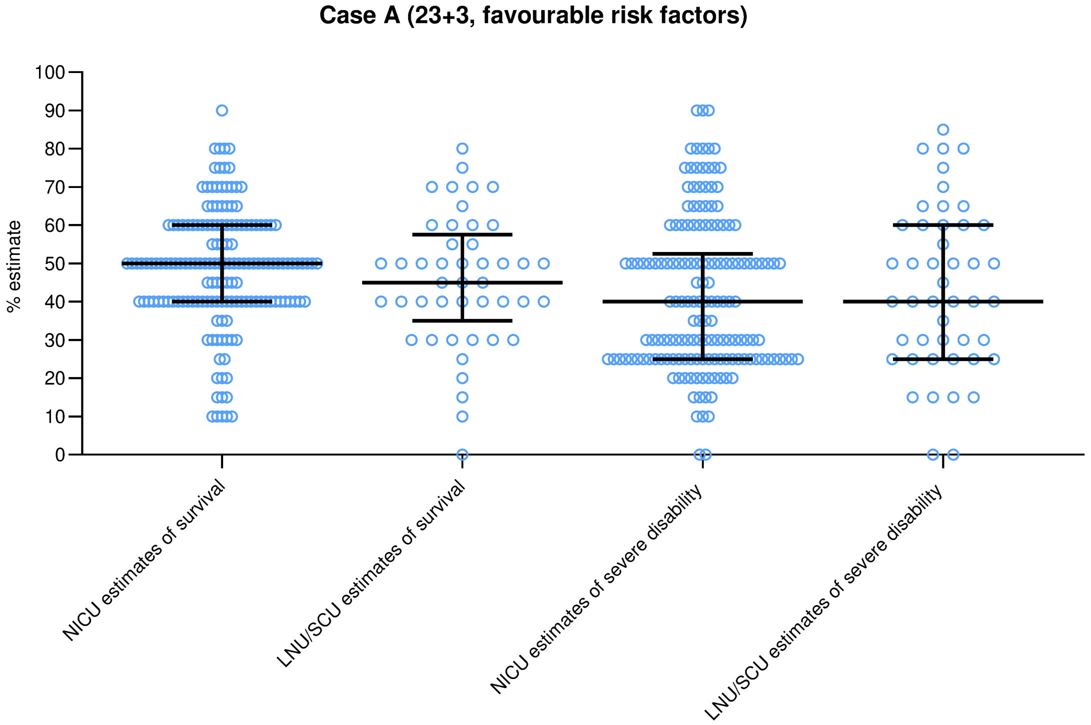
Case A (23+3, favourable risk factors)
% estimate
100
90
80
70
60
50
40
30
20
10
0
NICU estimates of survival
LNU/SCU estimates of survival
NICU estimates of severe disability
LNU/SCU estimates of severe disability

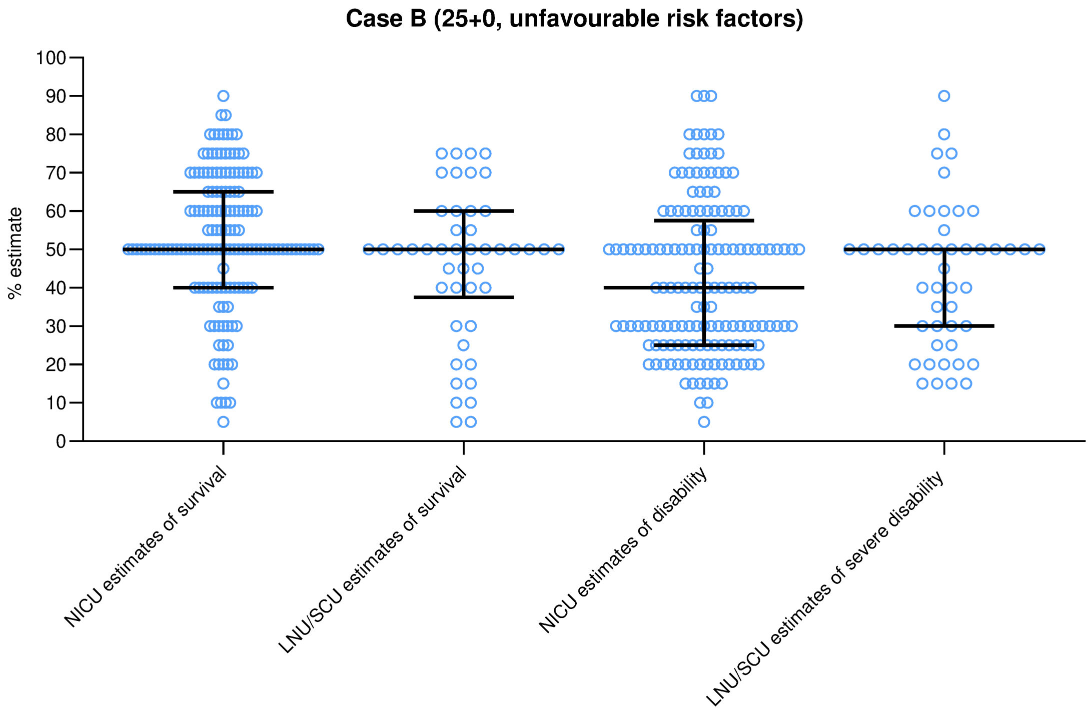
Case B (25+0, unfavourable risk factors)
% estimate
100
90
80
70
60
50
40
30
20
10
0
NICU estimates of survival
LNU/SCU estimates of survival
NICU estimates of disability
LNU/SCU estimates of severe disability

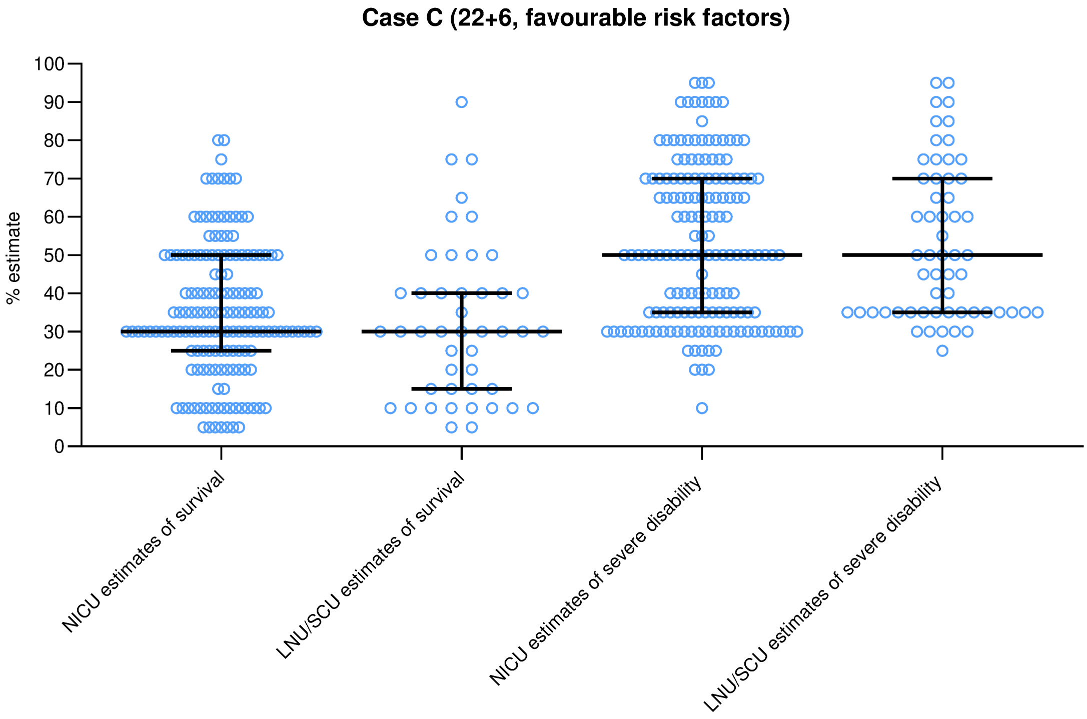
Case C (22+6, favourable risk factors)
% estimate
0
10
20
30
40
50
60
70
80
90
100
NICU estimates of survival
LNU/SCU estimates of survival
NICU estimates of severe disability
LNU/SCU estimates of severe disability

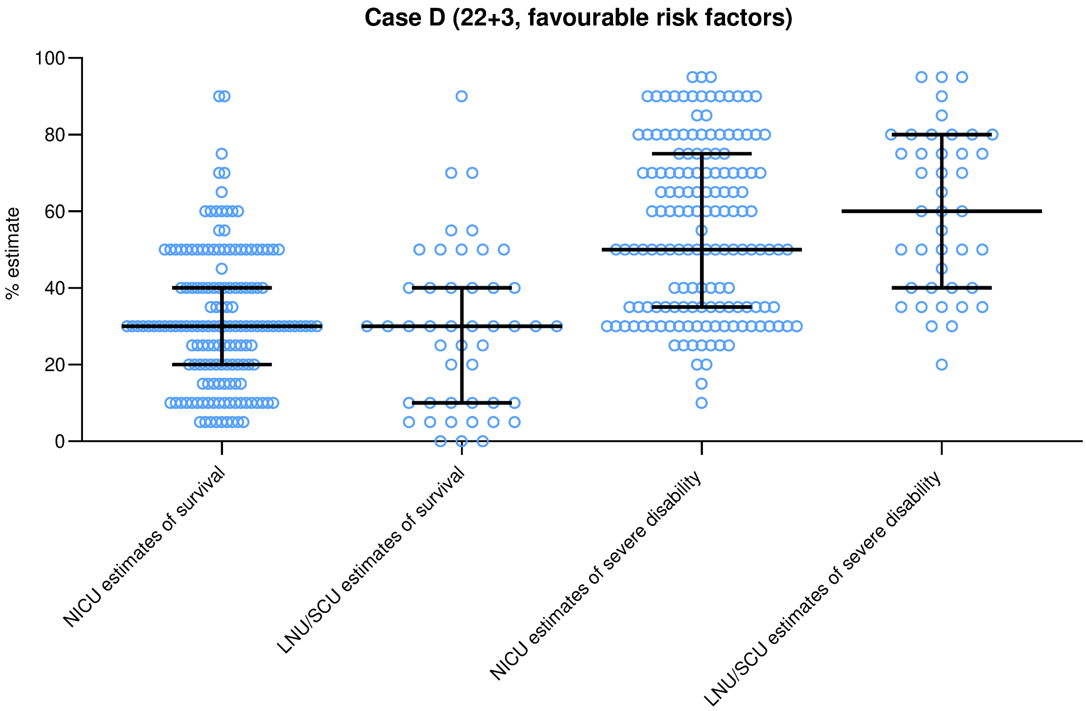
Case D (22+3, favourable risk factors)
% estimate
0
20
40
60
80
100
NICU estimates of survival
LNU/SCU estimates of survival
NICU estimates of severe disability
LNU/SCU estimates of severe disability

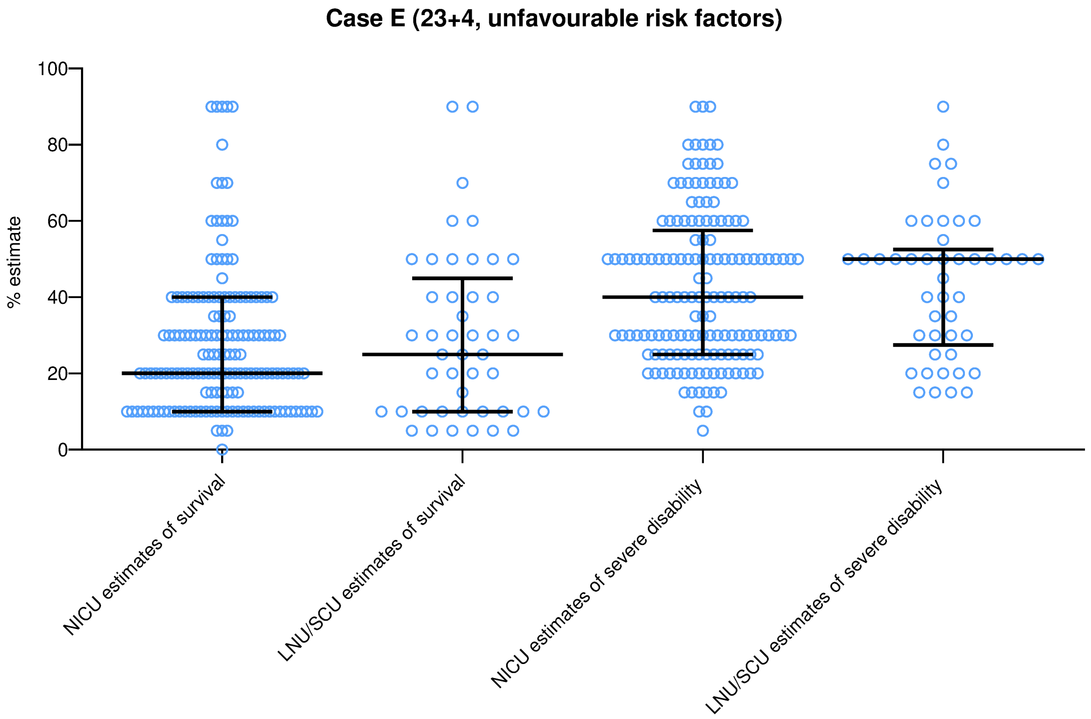


**Supplementary figure 2**: Dot plots showing respondents estimates of survival and severe disability in percentages by type of unit worked in. Each dot represents one respondent's estimates. The horizontal lines represent the median and interquartile range. There was no statistical difference found between type of unit worked in for both estimates of survival and severe disability (using Mann Whitney U test. Statistical significance was taken as p<0.005)

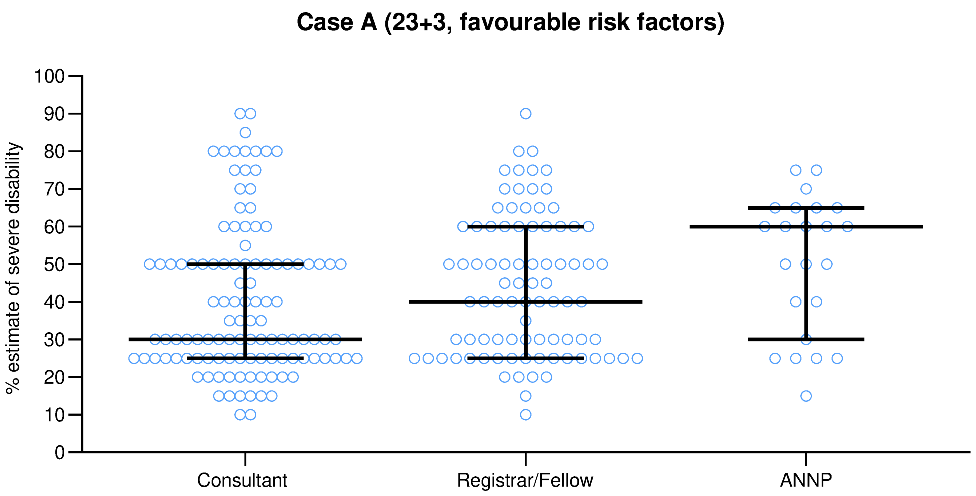
Case A (23+3, favourable risk factors)
% estimate of severe disability
0
10
20
30
40
50
60
70
80
90
100
Consultant
Registrar/Fellow
ANNP

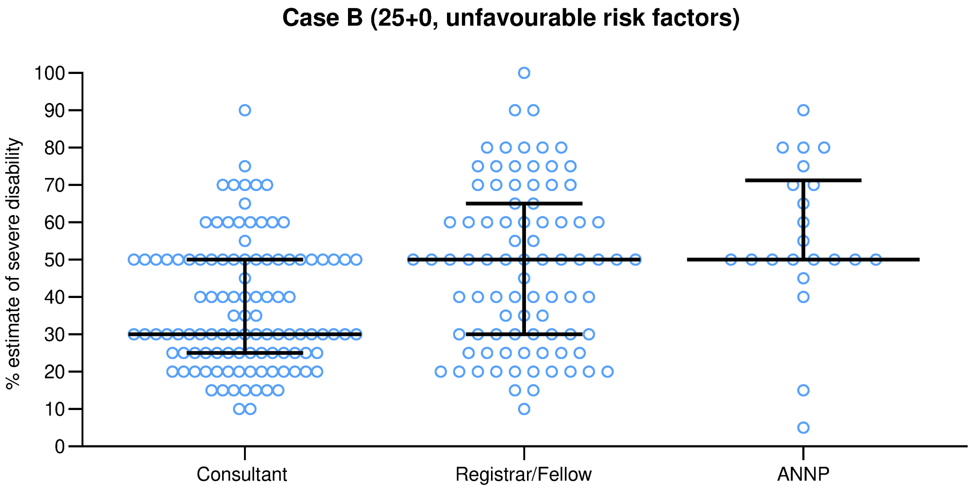
Case B (25+0, unfavourable risk factors)
% estimate of severe disability
0
10
20
30
40
50
60
70
80
90
100
Consultant
Registrar/Fellow
ANNP

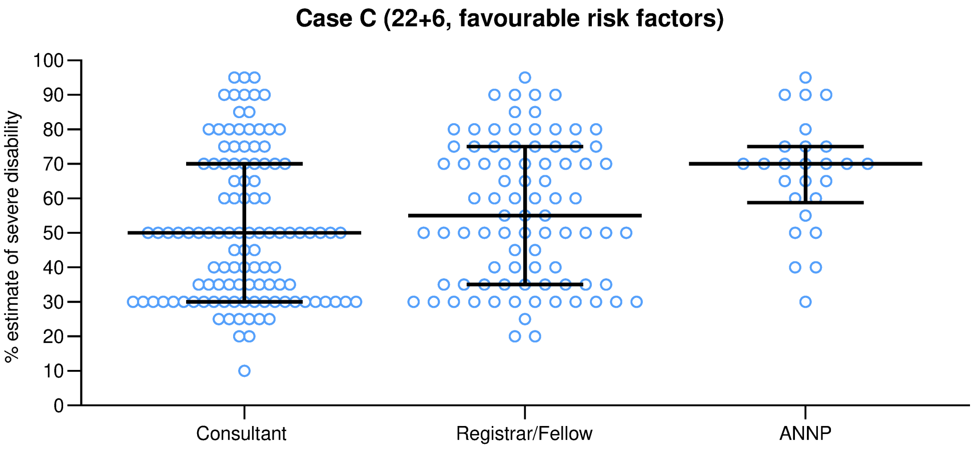
Case C (22+6, favourable risk factors)
% estimate of severe disability
0
10
20
30
40
50
60
70
80
90
100
Consultant
Registrar/Fellow
ANNP

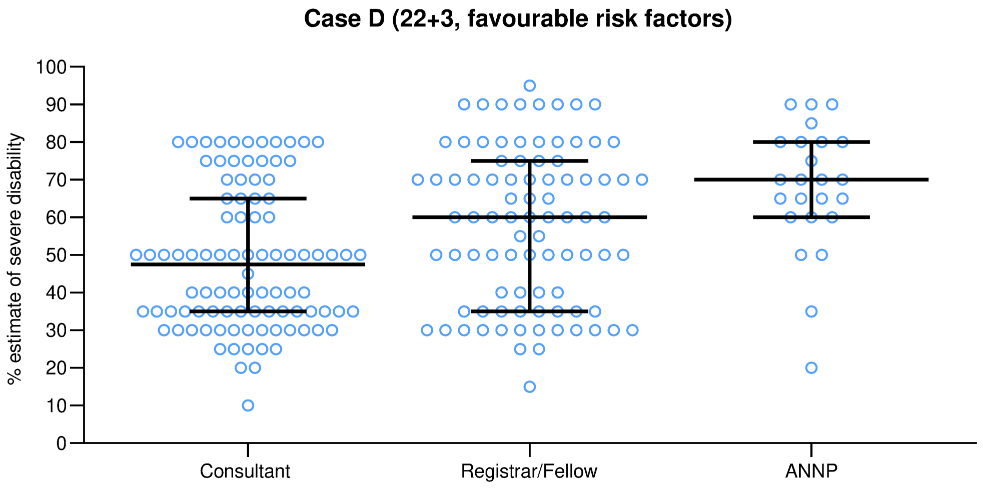


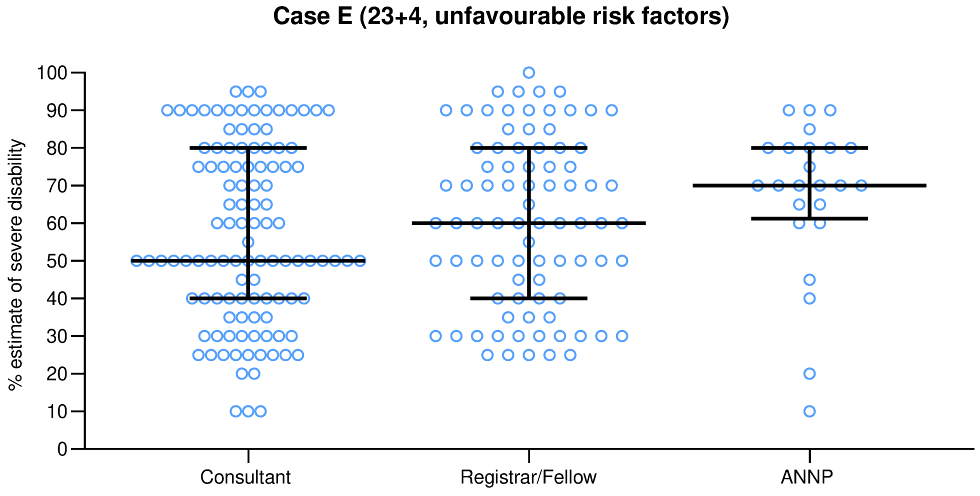


**Supplementary Figure 3:** Dot plots showing respondents estimates of severe disability in percentages by professional group. Each dot represents one respondent's estimates. Horizontal lines represent the median and interquartile range. There was statistically significant difference between estimates of consultants and registrars in cases B and D (case B; U=3381, p<0.0001 and case D; U=3135, p 0.004) and between consultants and ANNPs in cases B, C and D (case B; U=564, p <0.001, case C; U 826, p<0.001 and case D; U 531, p <0.001)
